# Rapid point-of-care (POC) testing for Hepatitis C antibodies in a very high prevalence setting: persons injecting drugs in Tallinn, Estonia

**DOI:** 10.1186/s12954-021-00485-5

**Published:** 2021-04-01

**Authors:** Anneli Uusküla, Ave Talu, Jürgen Rannap, David M. Barnes, Don Des Jarlais

**Affiliations:** 1 Department of Family Medicine and Public Health, University of Tartu, Ravila 19, 50411 Tartu, Estonia; 2 School of Global Public Health, New York University, 665 Broadway, New York, NY 10012 USA

**Keywords:** HCV testing, HCV antibody testing, HCV POC testing, HCV RNA testing, Persons who inject drugs, PWID, Estonia

## Abstract

**Background:**

Between December 2018 and January of 2019, we evaluated the accuracy of the point-of-care Hepatitis C (HCV) antibody test (POC; OraQuick HCV) used at a community-based needle and syringe exchange program serving persons who inject drugs in Tallinn, Estonia.

**Methods:**

We compared the results of screening for HCV antibodies by OraQuick (oral swab) and enzyme immunoassay (EIA; blood draw) and assessed test results implications in a high prevalence setting.

Findings

Of the 100 participants, 88 (88%) had reactive POC test results, and 93 were HCV antibody positive on EIA testing. Sensitivity, specificity and negative predictive value (NPV) for the POC assay with EIA as the relevant reference test were as follows: 94.6% (95% CI 90.0–99.2%), 100% and 58.3% (95% CI 30.4–86.2%). Of the 12 testing, HCV-negative with the POC only 7 (58.3%) were true negatives.

**Conclusions:**

Oral swab rapid testing HCV screening in this nonclinical setting was sensitive and specific but had unacceptably low NPV. In high prevalence settings, POC tests with high sensitivity and that directly measure HCV RNA may be warranted.

## Introduction

Drug use is central to sustaining hepatitis C virus (HCV) incidence in developed countries, with exposure to contaminated injection equipment among people who inject drugs (PWID) making them particularly vulnerable [1]. In addition, there is still significant ongoing transmission of HCV infection, with new injectors often becoming infected relatively rapidly [2]. Treatment of hepatitis C disease, and therefore treatment as prevention of HCV transmission, is now possible because of highly effective, tolerable, short-course interferon-free direct-acting antiviral therapies [3]. Cost-effectiveness models that incorporate the population prevention benefit suggest early treatment should be prioritized to PWID over other patient groups, unless chronic HCV prevalence and transmission is high in the population [4].

Currently, the main approach to reduce HCV disease burden is by increasing hepatitis C awareness in the public, including among higher risk groups, and health-care providers, and augmenting both screening and treatment of those found infected. Screening for HCV exposure entails HCV antibody testing (anti-HCV). Community-based testing, together with implementation of novel testing approaches in several settings, including drug services has been effective in reaching populations at higher risk for infection and hard‐to‐reach populations [5]. A sizable proportion (20–70%) [6–8] of drug users positive for HCV antibodies is likely to be RNA positive and be eligible for treatment with DAAs (HCV RNA positive).

The use of point-of-care (POC) tests to detect HCV antibodies has been implemented in a variety of settings in the USA and Australia, mostly at sites with HCV antibodies seropositivity < 30% (correctional centers [9], community-based settings (including needle and syringe exchange programs [10], urban STI clinics [11]) but not always (NSPs [12]).

Ease of sampling, performance and reading results, no requirement of cold chain or specialized equipment, and quick turnaround time make POC tests particularly suitable for community-based harm reduction service providers and other settings serving hard-to-reach populations [13]. If a key goal of HCV screening is to identify and expedite treatment to those in need, sensitivity and specificity might not be the only or most useful target metrics for testing algorithm development.

We assessed the performance and the potential implications of test results of the OraQuick point-of-care test for HCV antibodies from oral swab in comparison with the Abbott Anti-HCV assay by venipuncture among PWID in a community-based NSP in Tallinn, Estonia. Estonia experienced an epidemic of injecting drug use in the 1990s and in the decades, since the prevalence of HIV and HCV has been greater than 50% and 70%, respectively, among PWID [14, 15].

## Methods

We compared the results of screening by OraQuick (oral swab) and enzyme immunoassay (EIA; blood draw) in a high HCV and HIV prevalence setting.

Data were collected in a cross-sectional study of 100 PWID in Tallinn from December 2018 to January 2019 recruited through respondent driven sampling [16]. Potential participants were eligible for the study if they were at least 18 years of age, spoke Estonian or Russian, reported having injected in the previous two months, and were able and willing to provide informed consent.

Recruitment began with the non-random selection of ‘seeds’ (*n* = 8) purposefully selected (amongst PWID know to field team) to represent diverse PWID types (by age, gender, ethnicity, main type of drug used and HIV status). After they had participated in the study, subjects were provided with coupons for recruiting up to three of their peers (other persons who inject drugs). Coupons were uniquely coded to link participants to their survey responses and to biological specimens, and for monitoring who recruited whom. Participants who completed the study received a primary incentive (a grocery store voucher with the value of 10 euros) for participation in the study and a secondary incentive (a grocery store voucher with the value of 5 euros) for each peer recruited (peers had come to study site, be eligible and complete study procedures for recruiter to receive the incentive). We used an interviewer-administered questionnaire that elicited information on respondents’ demographics, injection drug use, testing and treatment for HIV and HCV.

### Testing

Study subjects who consented to participate provided an oral fluid specimen via a mouth swab for the POC HCV antibody test (OraQuick® HCV Rapid Antibody Test, OraSure Technologies Inc., Bethlehem, PA, USA). A study nurse collected the oral fluid specimen after pre-test counselling. All study staff were trained on the use of POC tests by representatives of the OraQuick distributor in the USA. Training also included review of data collection, specimen handling, test reading and interpretation. The testing was performed according to the manufacturer's instructions using oral specimens.

A test was interpreted as invalid if a control line was missing or broken, as negative if a control line was present (regardless of intensity) with no corresponding test line, and positive if both control and test lines were present. Results were determined visually after 20 to 40 min and were provided to participants on-site along with face-to-face post-test counselling.

All study subjects also underwent venipuncture by an experienced nurse. Venous blood was collected from participants and tested using 4th generation antigen/antibody test for HIV (ADVIA Centaur CHIV Ag/Ab Combo, Siemens Healthcare Diagnostic, Germany; all positive samples confirmed by Western blot (WB; NEW LAV BLOT I and NEW LAV BLOT II, Bio-Rad Laboratories Inc, Hercules, California, USA) and anti-HCV antibodies test (ARCHITECT Anti-HCV, Abbott Diagnostics, Abbott Park, IL, USA) (the reference test).

All participants were scheduled for a follow-up visit one week after their POC test to learn the results from their venous blood tests. Participants who completed the study received a primary incentive (a 10-euro grocery store voucher) and a secondary incentive (a 5-euro grocery store voucher) for each peer they recruited who enrolled in the study. Trained counsellors provided pre- and post-test counselling to study participants.

Ethical approval for the study was obtained from the Ethics Review Board of the University of Tartu, Estonia and from the Mount Sinai Beth Israel Medical Center Institutional Review Board in New York City, NY, USA. Written informed consent was secured from all participants.

### Data analysis

We first compared the primary data (the crude estimates of sample proportions) to RDS weighted data. The data collected in the process of recruiting the study subjects (i.e., the number of potential participants that the respondent knew within the target population and the coupon numbers of each respondent and his/her recruiter from the recruiting coupons) were used to derive RDS sequential sampling estimates for the mean value or the prevalence (with 95% CIs) for the variables of interest (RDS weighting). For almost all variables, there was little difference between the weighted and unweighted values (less than 5%). We therefore used the unweighted values in order to facilitate comparisons with other PWID studies that had not used RDS recruitment.

Results from the rapid POC anti-HCV test were compared with results from the conventional venous anti-HCV screening EIA test. In a recent systematic review of the studies assessing diagnostic accuracy of available rapid diagnostic tests and laboratory based EIA assays in detecting antibodies to HCV, 20% of studies were using enzyme-or chemiluminescence immune assays as a reference standard [17].

Given that we compared one screening assay to another screening assays (and not to immunoblot or PCR test), the tests performance characteristics are relative but not absolute measures of sensitivity, specificity and predictive values.

Sensitivity, specificity and predictive values, with their corresponding 95% confidence intervals, were calculated using the anti-HCV screening EIA as a standard reference. Sensitivity was defined as the percentage of reference test–positive specimens identified as positive by the POC test. Specificity was defined as the percentage of reference test–negative specimens identified as negative by the POC test. To assess the implications of test results for this setting, we calculated the positive predictive value (PPV; the percentage of POC-test positive specimens identified as positive by the reference test) and negative predictive value (NPV; the percentage of POC-test negative specimens identified as negative by the reference test). Further, we calculated the expected NPVs to quantify the probability that a person who is truly seropositive will be misclassified as negative (1-NPV) for tests with varying sensitivity (and 100% specificity) across a range of transmission intensities, represented by different levels of seroprevalence.

Data analysis was performed using R (version 3.5.1).

## Results

A total of 100 participants completed the study. The median age of participants was 36 (SD 6.8) years (interquartile range 31–41 years) and the majority (64%) were male. Ninety participants (90%) reported ever having a hepatitis C test before their study participation, and 65.5% (59/90) had been tested within the past 12 months. Two-thirds (*n* = 65) reported a previous HCV positive test, and of these, 19 (29.2%) reported having received HCV treatment.

Of the 100 participants who completed the POC antibody test, 88 (88%) had reactive results, 93 (93%) were HCV antibody positive on EIA testing, and 55 (55%) were HIV-infected. The HCV-HIV co-infection rate was 55%.

Sensitivity and specificity for the POC assay were as follows: 94.6% (95% CI 90.0–99.2%) for sensitivity; 100% for specificity and positive predictive value; and 58.3% (95% CI 30.4–86.2%) for NPV. The NPV results indicate that of those testing HCV-negative with the POC, only 58.3% (7/12) were true negatives (i.e., whose HCV reference test results were also negative).

If those who tested negative on POC (*n* = 12), but with actual HCV seropositivity of 41.6% (5/12), had been retested with another POC test (with sensitivity and specificity with minimum of 95%), the resulting NPV would have been 96.4%. This considerably higher NPV underscores the extent to which underlying prevalence affects NPV results, specifically that when using a test with high sensitivity and specificity, NPV decreases as underlying prevalence increases.

**Fig. 1:**
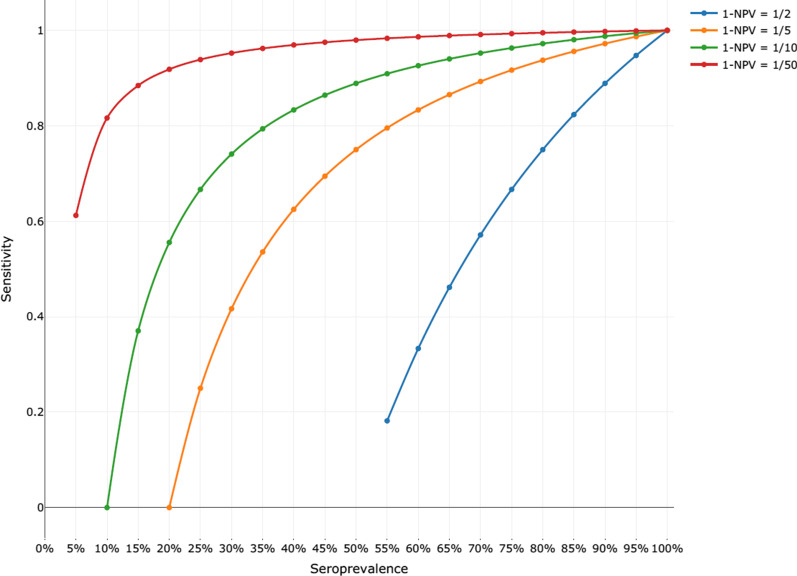
Effect of assay sensitivity and seroprevalence on probability of misclassification (1-NPV: probability that a person who tests negative is truly seropositive)

Figure 1 illustrates the minimum sensitivity that would be required in an assay (with specificity of 100%) to ensure a probability of misclassification (1-NPV) of a given value (or less), for a range of transmission settings, represented by different levels of seroprevalence among study subjects.

## Discussion

To date, there have been limited data to guide the selection of optimal testing strategies for viral hepatitis in different epidemic settings. Our study assessed the performance of a single POC HCV test and the implications of test results at a non-clinical site in a high seroprevalence setting. Our study documented 94.6% sensitivity and 100% specificity when comparing an oral HCV antibody POC test with a blood reference test results that are consistent with data from a meta-analysis [17]. The test has the major advantages of not requiring a blood, with the chance of exposure to infectious material. It is also a quick test, subjects can wait for their result rather than having to return for results at a later date.

However, predictive values, because they are sensitive to prevalence, are more appropriate and informative in actual screening contexts. Positive and negative predictive values are directly related to the prevalence of the disease in the population. Assuming all other factors remain constant, the PPV increases and the NPV decreases with increasing prevalence. Therefore, rather than solely focusing on sensitivity and specificity of the test, determining acceptable PPVs and NPVs is warranted, and subsequently determining the minimum sensitivity and specificity for different transmission settings that would achieve the target PPVs and NPVs. In low-prevalence settings, it is possible to achieve a NPV of more than 90% (10% of individuals who test seronegative will be misclassified, thereby precluding treatment) with screening tests across a range of sensitivities. By contrast, in settings with moderate or high seroprevalence, higher sensitivity is required to achieve a NPV of 90%. Importantly, the estimates of HCV antibody prevalence among PWID exceed 50% in most developed countries [18].

For non-clinical settings, there are three possible HIV point-of-care testing algorithms (single rapid test with immediate linkage to clinical provider if initial test is reactive; single rapid test followed by laboratory-based follow-up testing if initial test is reactive; and single rapid test immediately followed by a second rapid test on-site if initial test is reactive) [18]. Multi-test HIV screening via successive [19, 20] or simultaneous [21] rapid tests/POC algorithms have been adopted in research to maximize the specificity and positive predictive value. Two successive POC tests have lower costs than simultaneous POC tests [22]. For HCV screening, the POC testing algorithms are less well developed. Clinical settings and conventional tests (serological assays) were over-represented in both a recent review of HCV diagnostic algorithms [23] and a recent modeling study [24], shedding insufficient light on optimal algorithms in non-clinical settings using POC testing.

Testing strategy (including the type of test, biological material to be used, and methods for collection) needs to be tailored to the setting. POC testing can be useful to increase the number of subjects tested and linked with care and treatment in clinical settings [28, 29] and has proven to be specifically suitable for PWID [30, 31]. POC testing enables expanding testing options for PWID whose veins are heavily damaged and in settings where phlebotomy or laboratory services cannot be provided (e.g., in a mobile harm reduction van), thereby increasing the likelihood that prevention counselling and referrals for follow-up can be provided expeditiously [32]. However, in a high prevalence setting, even using an anti-HCV screening test with high sensitivity will lead to sizable misclassification. Using HCV RNA tests (such as POC HCV RNA tests) or HCV core antigen test [33] for HCV screening in high prevalence setting is well a justified alternative. The benefit of HCV RNA test lies not only in high sensitivity and specificity but importantly, in lower (than for anti-HCV tests) pre-test positivity (prevalence), and though with a high NPV. Sizable proportions of anti-HCV positive PWID are HCV RNA negative [34, 35] (due to the natural course or treatment of the HCV infection) [36, 37]. POC anti-HCV testing might be useful to obtain a preliminary estimate of HCV seroprevalence to inform testing strategy for a full study. If prevalence is low to moderate, POC anti-HCV testing might be worthwhile. Clearly, POC testing and self-sampling testing strategies warrant further research, especially in light of the COVID-19 epidemic.

## Limitations

As for any single-centre study with a modest sample size, further confirmation by an independent study is desirable. Given high HCV antibody prevalence, we were unable to stratify the analysis by HIV status (all HCV antibodies positives were also HIV positive). Several studies have documented compromised HCV antibody test performance among HIV-infected individuals [25, 26], including in the context of POC testing [27]]. Last, but not least, the reference standard used in this study is subject to some misclassification error that may affect the apparent diagnostic performance of the POC tests being evaluated.

## Conclusions

A key public health objective of an effective testing strategy is to identify all individuals who would benefit from treatment. However, the testing strategy and test to be used should be tailored to each context to achieve the desired impact on HCV elimination. In high prevalence settings and in high transmission risk groups, tests with very high sensitivity (a strategy that prioritizes a high NPV-minimal false negatives) may be preferable even if the PPV is suboptimal (some false positives).

## Data Availability

The data include extremely sensitive information, such as HIV status and illegal activity. Per the grant proposals, the IRB approvals and the Informed Consents, even anonymized individual data cannot be made publicly available. Access to the data is possible through formal Data Use Agreements between the two institutions with legal responsibility for maintaining confidentiality of the data (the University of Tartu and New York University) and the institution employing the research who desires access to the data. Investigators interested in entering into a Data Use Agreement to obtain access to the data should contact Drs. Uusküla and Des Jarlais.
